# Acute Fetal Demise with First Trimester Maternal Infection Resulting from *Listeria monocytogenes* in a Nonhuman Primate Model

**DOI:** 10.1128/mBio.01938-16

**Published:** 2017-02-21

**Authors:** Bryce Wolfe, Gregory J. Wiepz, Michele Schotzko, Gennadiy I. Bondarenko, Maureen Durning, Heather A. Simmons, Andres Mejia, Nancy G. Faith, Emmanuel Sampene, Marulasiddappa Suresh, Sophia Kathariou, Charles J. Czuprynski, Thaddeus G. Golos

**Affiliations:** a Wisconsin National Primate Research Center, University of Wisconsin—Madison, Madison, Wisconsin, USA; b Department of Pathology and Laboratory Medicine, University of Wisconsin—Madison, Madison, Wisconsin, USA; c Department of Comparative Biosciences, University of Wisconsin—Madison, Madison, Wisconsin, USA; d Department of Pathobiological Sciences, University of Wisconsin—Madison, Madison, Wisconsin, USA; e Department of Biostatistics and Medical Informatics, University of Wisconsin—Madison, Madison, Wisconsin, USA; f Department of Obstetrics and Gynecology, University of Wisconsin—Madison, Madison, Wisconsin, USA; g Department of Food, Bioprocessing and Nutrition Sciences, North Carolina State University, Raleigh, North Carolina, USA; Umea University; Washington University School of Medicine

## Abstract

Infection with *Listeria monocytogenes* during pregnancy is associated with miscarriage, preterm birth, and neonatal complications, including sepsis and meningitis. While the risk of these conditions is thought to be greatest during the third trimester of pregnancy, the determinants of fetoplacental susceptibility to infection, the contribution of gestational age, and the *in vivo* progression of disease at the maternal-fetal interface are poorly understood. We developed a nonhuman primate model of listeriosis to better understand antecedents of adverse pregnancy outcomes in early pregnancy. Four pregnant cynomolgus macaques (*Macaca fascicularis*) received a single intragastric inoculation between days 36 and 46 of gestation with 10^7^ CFU of an *L. monocytogenes* strain isolated from a previous cluster of human listeriosis cases that resulted in adverse pregnancy outcomes. Fecal shedding, maternal bacteremia, and fetal demise were consistently noted within 7 to 13 days. Biopsy specimens of maternal liver, spleen, and lymph node displayed variable inflammation and relatively low bacterial burden. In comparison, we observed greater bacterial burden in the decidua and placenta and the highest burden in fetal tissues. Histopathology indicated vasculitis, fibrinoid necrosis, and thrombosis of the decidual spiral arteries, acute chorioamnionitis and villitis in the placenta, and hematogenous infection of the fetus. Vascular pathology suggests early impact of *L. monocytogenes* infection on spiral arteries in the decidua, which we hypothesize precipitates subsequent placentitis and fetal demise. These results demonstrate that *L. monocytogenes* tropism for the maternal reproductive tract results in infection of the decidua, placenta, and the fetus itself during the first trimester of pregnancy.

## IMPORTANCE

Although listeriosis is known to cause significant fetal morbidity and mortality, it is typically recognized in the third trimester of human pregnancy. Its impact on early pregnancy is poorly defined. Here we provide evidence that exposure to *L. monocytogenes* in the first trimester poses a greater risk of fetal loss than currently appreciated. Similarities in human and nonhuman primate placentation, physiology, and reproductive immunology make this work highly relevant to human pregnancy. We highlight the concept that the maternal immune response that protects the mother from serious disease is unable to protect the fetus, a concept relevant to classic TORCH (toxoplasmosis, other, rubella, cytomegalovirus, and herpes) infections and newly illuminated by current Zika virus outbreaks. Studies with this model, using the well-understood organism *L. monocytogenes*, will permit precise analysis of host-pathogen interactions at the maternal-fetal interface and have broad significance to both recognized and emerging infections in the setting of pregnancy.

## INTRODUCTION

*Listeria monocytogenes* is an environmentally ubiquitous bacterium that causes foodborne illness. While infection in healthy individuals is generally not associated with significant disease, it poses a substantial risk for specific populations, including immunocompromised, elderly, or pregnant individuals. Listeriosis during pregnancy is generally associated with a spectrum of adverse outcomes during the third trimester, including miscarriage, preterm labor, stillbirth, and neonatal infection (1). Inflammation of fetal membranes and placental villitis are noted in these cases (2), but the actual course of events at the maternal-fetal interface that cause fetal demise are poorly understood. In the clinic, human placental tissues generally are not available until after an adverse outcome, at which point fetal infection has progressed for an indeterminate period of time. *In vitro* studies with tissue explants from human first (3) or third (4) trimester placentas concluded that either extravillous trophoblasts (3) or syncytiotrophoblasts (4) are likely the primary route of placental infection. However, *in vitro* studies are limited in reproducing the physiological and immunological complexity at the maternal-fetal interface, and it is unclear how infection proceeds *in vivo*. Growing recognition of the impact of microbial infections on fetal well-being (e.g., Zika virus or TORCH [toxoplasmosis, other, rubella, cytomegalovirus, and herpes] infections) (5, 6) underscores the importance of gaining a better mechanistic understanding of such events. The aim of this study was to establish a nonhuman primate model to map the outcome of infection with *L. monocytogenes* during early pregnancy. Like humans, macaques and other nonhuman primates have villous hemochorial placentas and invasive extravillous fetal trophoblasts that actively remodel maternal spiral arteries in the decidua. Their similar endocrine, reproductive, and immune systems make nonhuman primates a highly relevant model for examining the interplay of maternal and fetal responses to infection during pregnancy. Remarkably, first trimester inoculation of cynomolgus macaques consistently resulted in maternal and fetal infection followed by fetal death within 7 to 13 days postinoculation. This contrasts with the general assumption that listeriosis is of greatest concern during the third trimester of pregnancy. Microbial analysis of collected tissues revealed that the decidua basalis and placental bed as well as the placenta had significant bacterial burden. These findings suggest that maternal infection affects both the placenta—the presumptive target of listeriosis during human pregnancy—and the maternal reproductive tract.

## RESULTS

### Physiological responses to *L. monocytogenes* inoculation.

Table S1 in the supplemental material summarizes the infection schedule, dose, and vital signs of each animal. Four cynomolgus macaques were inoculated in early gestation. Each animal received ~10^7^ CFU of *L. monocytogenes* in 10 ml of whipping cream through a soft intragastric feeding tube while under sedation. This strain of *L. monocytogenes* was associated with a listeriosis outbreak that resulted in adverse pregnancy outcomes in 11 pregnant women (7). Following inoculation, animals were observed to ensure bacteria were not lost due to emesis. Subsequently, animals were monitored for changes in behavior, defecation, and food intake. There were no notable behavioral changes associated with *L. monocytogenes* administration. Two of the animals that received *L. monocytogenes* had mild fever (body temperature of >38.89°C) in the days prior to fetal demise.

10.1128/mBio.01938-16.4 TABLE S1  Maternal physiological responses to inoculation. Reference intervals for adult female cynomolgus macaques: temperature, 35 to 37.78°C (95 to 100°F); systolic blood pressure, 84 to 130 mm Hg; mean arterial pressure, 81 to 113 mm Hg; diastolic blood pressure, 53 to 80 mm Hg. Download TABLE S1, DOCX file, 0.1 MB. Copyright © 2017 Wolfe et al. 2017 Wolfe et al. This content is distributed under the terms of the Creative Commons Attribution 4.0 International license.

### Fecal shedding.

**FIG 1:**
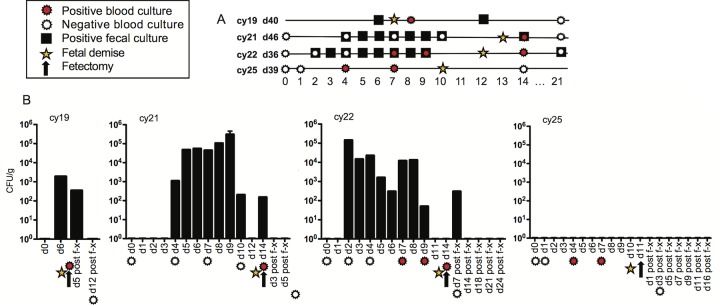
(A) Timeline of events and laboratory analyses for all 4 animals (ID no. cy19, cy21, cy22, and cy25) and key to symbols for events on the timeline. Positive blood cultures, negative blood cultures, positive fecal cultures, and estimated day of fetal demise are shown. Overlapping symbols indicate concurrent events. The gestation day at inoculation (d40, d46, d36, and d39) is given after each animal ID. (B) The bar graphs indicate fecal shedding of *L. monocytogenes* for individual animals. The *x* axis depicts the days postinoculation, with symbols indicating events as shown in panel A. The arrow on each graph indicates the day of surgical collection of fetal tissues and maternal biopsy specimens. The *y* axis quantifies fecal shedding in CFU per gram. The absence of a bar denotes that a sample was collected but no *L. monocytogenes* was detectable on that date.

*L. monocytogenes* was not detectable in any fecal samples prior to inoculation and was first detectable in the stool between 1 and 5 days postinoculation in 3 out of 4 animals. In 2 animals, there was intermittent shedding of bacteria with detection of colonies up to 3 weeks postinoculation (Fig. 1A and B). *L. monocytogenes* was not detected at any date in feces from one animal (cy25), although this animal had both bacteremia and fetal demise. Control animals did not shed *L. monocytogenes* at any time.

### Bacteremia.

Peripheral blood samples were collected to monitor bacteremia pre- and postinoculation. There was no detectable growth in preinoculation samples. Bacteremia was detectable in all animals in at least one sample within 4 to 14 days of inoculation (Fig. 1A and B).

### Hematology.

Complete blood cell counts were within reference ranges for female cynomolgus macaques, except for elevated numbers of monocytes near the day of surgery (see Fig. S1 in the supplemental material). One animal that received *L. monocytogenes* had elevated lymphocytes, and 2 of the 4 animals had elevated basophils in comparison to control animals, but there were no statistically significant differences between pre- and postinoculation levels of any leukocyte subset within individual animals. The differences observed among subjects most likely relate to individual variation in a genetically diverse animal model rather than response to infection.

10.1128/mBio.01938-16.1 FIG S1  Peripheral blood leukocyte absolute counts, determined by automated cell analyzer. The *x* axis presents the experimental day relative to day of inoculation, and the *y* axis depicts thousands of cells per microliter. Red lines indicate cell counts from infected animals and black lines indicate cell counts from control animals. The blue shaded area indicates the reference interval for each cell type in adult macaques. Download FIG S1, TIF file, 0.4 MB. Copyright © 2017 Wolfe et al. 2017 Wolfe et al. This content is distributed under the terms of the Creative Commons Attribution 4.0 International license.

### Pregnancy outcome.

Fetal demise occurred within 7 to 13 days postinfection in all four pregnancies. Figure 1 summarizes each animal’s gestation day at inoculation, postinfection fecal shedding of *L. monocytogenes*, peripheral blood bacteremia, day of surgical collection of tissues when loss of fetal heartbeat was detected by ultrasound, and estimated day of fetal death based on the date of the last documented fetal heartbeat and the degree of autolysis observed during histological examination.

### Tissue bacterial burden.

**FIG 2:**
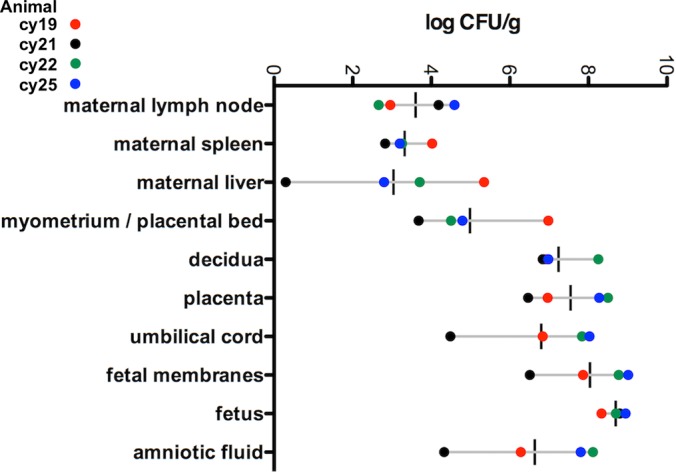
Maternal and fetal tissue burden of *L. monocytogenes*. The chart represents data from 4 animals. Tissues are shown on the *y* axis, and the *x* axis shows the CFU per gram on a log scale. The vertical black lines indicate the mean CFU per gram for each tissue, and horizontal gray lines indicate the range. Individual animals are noted in the legend.

Bacteriological analysis of maternal biopsy specimens and fetal tissue homogenates demonstrated remarkable similarity across all pregnancies (Fig. 2), with average bacterial burdens of <10^5^ CFU/g in maternal nonreproductive tissues of lymph node, spleen, and liver, 2 × 10^6^ CFU/g in placental bed/myometrium, 2 × 10^7^ CFU/g in umbilical cord and amniotic fluid, 1 × 10^8^ CFU/g in decidua and placenta, and >10^8^ CFU/g in fetal tissues.

### Control inoculations.

Two dams in the first trimester of pregnancy were fed 10 ml of whipping cream without *L. monocytogenes* to serve as gestational age-matched negative controls and were monitored by the same parameters as *Listeria*-inoculated animals. Uterotomy was performed at gestation days 51 (cy27) and 55 (cy26) for removal of the fetoplacental unit. Maternal and fetal tissues were processed in the same manner as experimental animals. *L. monocytogenes* was not detected in feces, blood, maternal tissues, or fetal tissues.

### Gross examination.

At surgery, the placental discs from infected animals were noted to be mottled yellow-white to red-purple with focal to multifocal areas of hemorrhage and coalescing areas of pallor (see Fig. S2 in the supplemental material). Umbilical cords were edematous, with vessels visible through the serosal surface. Fetal tissues were variably edematous and fragile, especially the liver. The lungs were mildly mottled. The fetus of one animal, cy19, had mild to moderate fetal tissue autolysis compatible with fetal demise approximately 18 to 24 h prior to collection. No pathologies were noted in control animals.

10.1128/mBio.01938-16.2 FIG S2  Gross examination of the placenta. (A) Listeria-positive first trimester conceptus with cloudy amnionic membranes (*), a large primary disc (1°), and smaller secondary disc (2°). Both discs have “dry” roughened decidual (maternal) surfaces with diffuse pallor and multifocal hemorrhage. (B) Normal first trimester conceptus with translucent amnionic membranes (*) allowing visualization of the fetus, a large primary disc (1°), and slightly smaller secondary disc (2°). Download FIG S2, TIF file, 1.5 MB. Copyright © 2017 Wolfe et al. 2017 Wolfe et al. This content is distributed under the terms of the Creative Commons Attribution 4.0 International license.

### Histopathology at the maternal-fetal interface.

**FIG 3:**
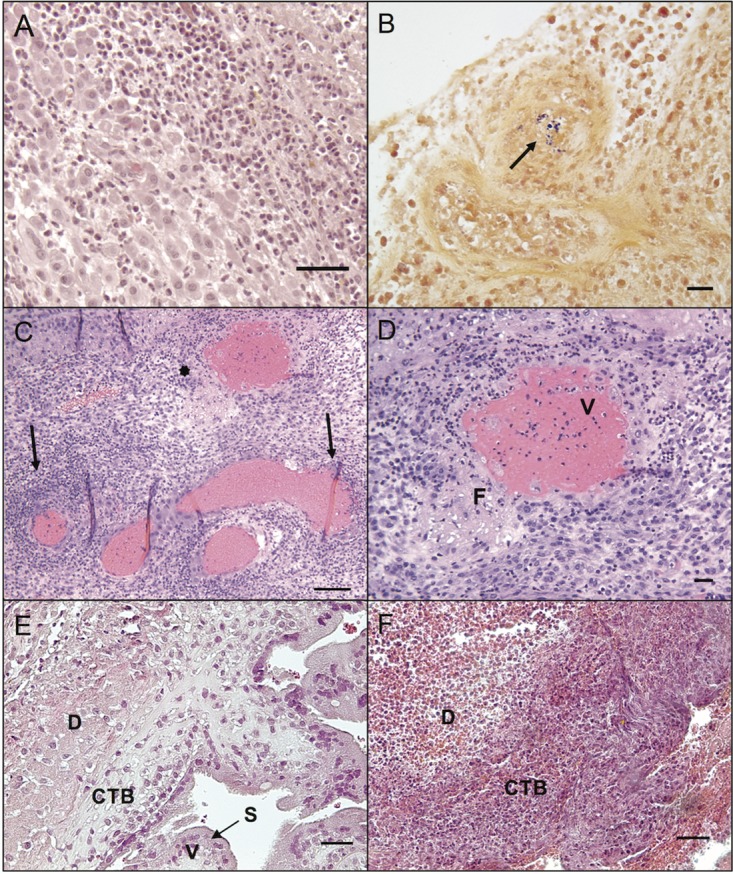
Histopathology of placental bed and decidua in *L. monocytogenes*-infected animals. (A) Diffuse suppurative deciduitis. H&E, 200-μm scale bar. (B) Intravascular Gram-positive rods in maternal decidual spiral artery (arrow). Gram stain, 200-μm scale bar. (C) Diffuse suppurative deciduitis with vasculitis of the maternal spiral arteries. Arrows indicate perivascular inflammation. An asterisk denotes the vessel shown in panel D. H&E, 400-μm scale bar. (D) Higher magnification of the spiral artery indicated by the asterisk in panel C with transmural suppurative vasculitis (V) and fibrinoid necrosis (F). H&E, 200-μm scale bar. (E) Normal decidua (D) with cytotrophoblast shell (CTB), placental villi (V), and intact syncytial layer (S) from a control animal. H&E, 400-μm scale bar. (F) *Listeria*-infected decidua with suppurative necrosis, diffuse edema, and syncytial degeneration. H&E, 400-μm scale bar.

**FIG 4:**
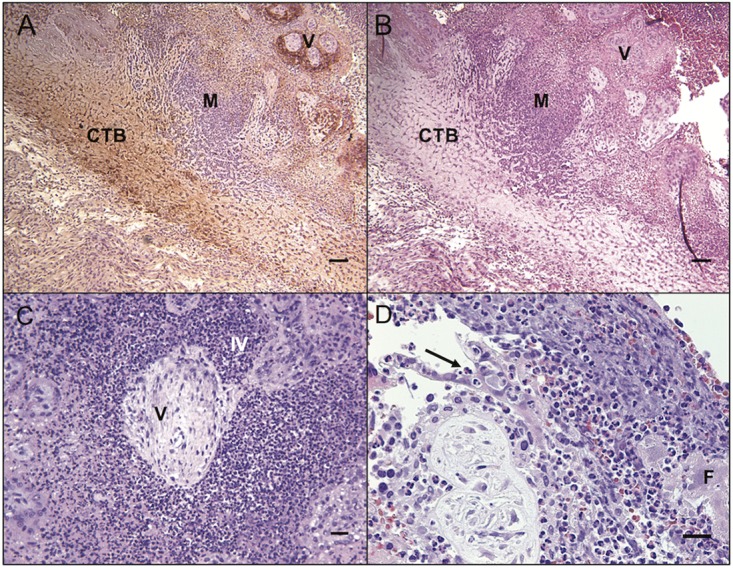
Histopathology of placenta in *L. monocytogenes*-infected animals. (A) Immunohistochemistry (IHC) staining for cytokeratin demonstrating fetal trophoblasts (brown precipitate) in the cytotrophoblastic shell (CTB) with microabscesses (M) near fetal villi (V). IHC stain, 400-μm scale bar. (B) Cytotrophoblastic shell (CTB) with microabscesses (M) near fetal villi (V). H&E, 400-μm scale bar. (C) Placental villous necrosis (V) with loss of syncytial layer and suppurative intervillositis (IV) in the placenta. H&E, 200-μm scale bar. (D) Diffuse suppurative placentitis with a representative neutrophil within the sloughed syncytial layer (arrow) and fibrin deposition (F) in the intervillous space. H&E, 200-μm scale bar.

**FIG 5:**
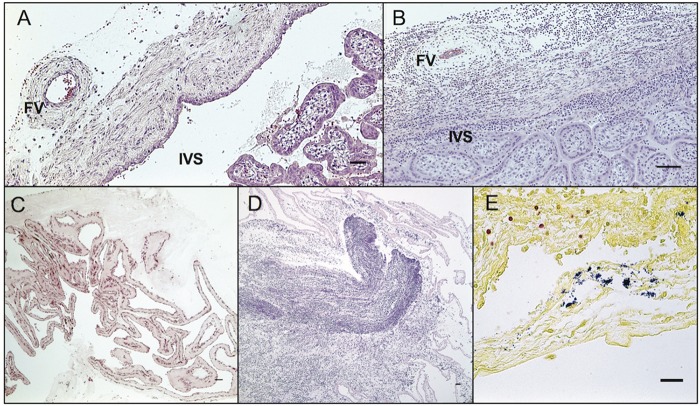
Fetal membranes and umbilical cord histology. (A) Normal chorionic plate with fetal vessel (FV) and clear intervillous space (IVS) from a control animal. H&E, 400-μm scale bar. (B) Chorioamnionitis with vasculitis of the fetal vessel (FV) and intervillositis (IVS). H&E, 400-μm scale bar. (C) Normal fetal membranes from a control animal. H&E, 600-μm scale bar. (D) *Listeria*-infected fetal membranes with edema and marked suppurative infiltrate. H&E, 600-μm scale bar. (E) Umbilical cord with Gram-positive rods (blue) within the vascular lumen, adherent to the residual endothelium, within the vascular wall, and extending into the adjacent perivascular stoma. Nuclei are stained red. Gram stain, 200-μm scale bar.

Placental bed and decidual specimens demonstrated significantly greater bacterial burden than other maternal tissues, which correlated with histologic lesions of multifocal suppurative endometritis and deciduitis (Fig. 3A) with sporadic Gram-positive intralesional bacteria (Fig. 3B). Decidual tissues from all animals demonstrated vascular pathology on histologic examination (Fig. 3C). Decidual spiral arteries, normally remodeled by endovascular trophoblasts, demonstrated vasculitis, necrotic fibrosis, and thrombosis highly atypical for early pregnancy (8) (Fig. 3D). Gram-positive rods were observed within maternal spiral arteries (Fig. 3B). Suppurative necrosis and edema within the decidua basalis were marked, in contrast to control tissues (Fig. 3E and F). The cytotrophoblastic shell that anchors the placenta and demarcates the boundary between maternal decidua basalis and fetal villi (9) demonstrated neutrophilic infiltration, multifocal necrosis of anchoring villi and syncytium, and multiple microabscesses (Fig. 4A and B). Histologic examination of placental specimens included hematoxylin and eosin (H&E), Gram stain, and *Listeria*-specific immunofluorescence. There was mild to severe multifocal necrosuppurative villitis, intervillositis (Fig. 4C and D), and vasculitis with necrosis of villous vessels and intralesional bacteria. Fetal membranes contained Gram-positive and listeria O antigen-positive rods with severe diffuse necrotizing and suppurative chorioamnionitis with moderate edema (Fig. 5A to D; see Fig. S3C in the supplemental material). The umbilical cord had moderate diffuse edema, mild multifocal neutrophilic and plasmacytic vasculitis and perivasculitis, thrombophlebitis, and mild multifocal vascular fibrinoid necrosis with intralesional bacteria (Fig. 5E).

10.1128/mBio.01938-16.3 FIG S3  Immunofluorescent staining for *L. monocytogenes* in the placenta and fetal membranes. (A) Bacteria (green) and nuclei (blue) in the placental chorionic villi (V). IHC, 200-μm scale bar. (B) Placenta immunostaining with a nonspecific isotype control, 200-μm scale bar. (C) Bacteria (green) and nuclei (blue) in the fetal membranes. IHC, 100-μm scale bar. (D) Fetal membranes with a nonspecific isotype control. 100-μm scale bar. Download FIG S3, TIF file, 2.7 MB. Copyright © 2017 Wolfe et al. 2017 Wolfe et al. This content is distributed under the terms of the Creative Commons Attribution 4.0 International license.

### Maternal extrauterine histopathology.

Maternal extrauterine tissue biopsy specimens demonstrated variable and relatively low levels of infection as determined by bacteriological assay (Fig. 2). Tissue CFU per gram ranged between 0 and 10^5^ in liver and between 10^2^ and 10^4^ in lymph node and spleen. cy19 and cy22 had moderate diffuse neutrophilia in the red pulp of the maternal spleen and mild diffuse suppurative hepatitis with single-cell necrosis, focal vasculitis, and mild diffuse glycogen vacuolization. cy25 had mild diffuse suppurative splenitis and mild suppurative and lymphoeosinophilic hepatitis.

## DISCUSSION

While it is recognized that adverse pregnancy outcomes associated with maternal listeriosis typically include placental infection, the precise pathogenesis of placental infection and subsequent fetal infection remains incompletely described. This study demonstrates that within 7 to 13 days following first trimester intragastric inoculation, there is modest infection of maternal extrauterine tissues with extraordinarily high numbers of *L. monocytogenes* in the decidua and placenta. In addition, degenerative changes in spiral arteries were associated with the implantation site within 8 days postinoculation. These results reveal a remarkable tropism, attack rate, and consistent pathophysiology in first trimester listeriosis.

It was previously reported that adverse outcomes occurred in approximately 30% of macaques infected with *L. monocytogenes* at approximately day 110 of gestation (10). Animals with stillbirth shed *L. monocytogenes* from the gastrointestinal tract for a longer period of time than animals with normal birth. These authors estimated that the fetal 50% lethal dose (LD_50_) was 1 × 10^7^ organisms. Consistent with our study, these authors did not observe outward signs of maternal illness or significant changes in blood cell counts. In contrast to these third trimester experimental infections, all four animals infected in the first trimester of pregnancy in the present study rapidly progressed to fetal demise. Three animals displayed fecal shedding of *L. monocytogenes*, and all animals had positive blood cultures in conjunction with fetal demise. It is possible that the strain of *L. monocytogenes* employed in the present study is more virulent than strains used in prior macaque studies. However, our observation of unremarkable pregnancy progression and lack of fetal or placental infection following a third trimester (day 110) inoculation with this strain suggests that gestational age may influence maternal-fetal susceptibility to infection. Thus, it appears that the maternal-fetal interface in early pregnancy in the macaque is exquisitely sensitive to listeriosis.

Only a few reports of pregnancy outcome in nonhuman primates with listeriosis are available (11), and none examined early gestational experimental infection. Similarly, there are few reports of pregnancy outcome in early gestation with human cases of listeriosis (1, 12, 13). Because the majority of case reports come from third trimester and neonatal infections, listeriosis is often characterized as a concern for late pregnancy (14, 15). We suggest that the number of first trimester miscarriages due to infection by *L. monocytogenes* is underreported because maternal listeriosis is often asymptomatic and unlikely to raise suspicion of infection (16). As maternal complications and fetal loss in late pregnancy will require medical attention, they are more likely to be reported.

The ability to study early pregnancy loss in a nonhuman primate model with placentation and gestation highly similar to those of humans is invaluable. Collection of discrete tissue samples and monitoring of maternal and fetal biomarkers throughout pregnancy are not possible in human studies, and the prolonged incubation periods seen in human outbreaks (17, 18) make it difficult to accurately determine exposure dose or timing of infection. Controlling these parameters in a nonhuman primate model allows precise monitoring of disease progression. Subsequent studies to characterize immune cell activation at the maternal-fetal interface, ontogeny of pathogenesis, and consequences of reexposure to *L. monocytogenes* will shed additional light on mechanisms of fetal loss in listeriosis.

How *L. monocytogenes* enters the placenta remains incompletely understood. As a foodborne pathogen, *L. monocytogenes* trafficks from the maternal gastrointestinal tract to maternal organs, including the spleen, liver, and lymph nodes, before colonizing the reproductive tract. Nonpregnant women without comorbidities are as resistant to infection as other healthy individuals; however, even healthy pregnant women have an increased risk of listeriosis (19, 20). Bakardjiev et al. demonstrated in a guinea pig model that only a few bacteria need reach the placenta to proliferate and then disseminate back to the maternal organs, because the placenta provides an immune-privileged reservoir for replication (21). Placentitis is a hallmark of listeriosis, but it is possible that the placenta may be an organ of collateral damage as well as a proximal target of *L. monocytogenes*. Poulsen et al. previously reported that LM2203 colonizes the uteri of nonpregnant as well as pregnant mice and suggested that fetal loss may be a consequence of preferential trafficking to the uterus (22). Early in gestation, fetus-derived extravillous trophoblast cells invade the decidua and remodel the maternal vasculature to ensure a sufficient exchange of nutrients and oxygen between mother and fetus. These structural changes may facilitate colonization of the uterus and potentially give entry to the fetal vasculature that underlies the syncytiotrophoblast, which itself has been found to be restrictive to infection (23, 24). As in humans, nonhuman primate placentas are comprised of cotyledons, or lobules, which contain a main stem villus with its branches (25). Remarkably, in animal cy19, the placental cotyledon associated with an individual disrupted spiral artery (Fig. 3C) had concomitant villitis.

**FIG 6:**
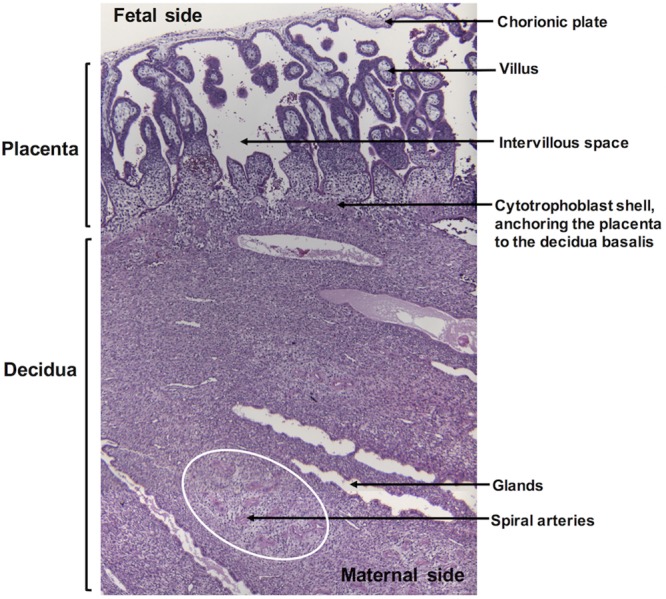
Overview of the macaque maternal-fetal interface in the first trimester of pregnancy. Specific structures of the placenta and decidua are highlighted.

One means of bacterial entry into the placenta may be through degeneration of the syncytiotrophoblast layer during the acute inflammatory response to *L. monocytogenes* at the maternal-fetal interface. The nonpregnant endometrium and pregnant decidua contain a resident maternal immune cell population that has been found to regulate *L. monocytogenes* infection in murine models of early pregnancy (26). In mice, bacterial burden was associated with restriction of cytotoxic T cells and macrophages from the decidua, suggesting that maternal immune tolerance toward the fetus may allow for unchecked bacterial proliferation. A distinct advantage of this nonhuman primate model is that macaque and human decidua contain highly similar subsets of NK cells, macrophages, and regulatory T cells, which are thought to play crucial roles in remodeling of decidual spiral arteries and maintenance of maternal tolerance toward fetal tissues (27–30). Given the extensive neutrophilic infiltrate noted in our study, we propose that *L. monocytogenes* elicits a strong proinflammatory response in the nonhuman primate decidua that resolves maternal infection but results in damage to the integrity of the maternal-fetal interface, thus allowing *L. monocytogenes* to traverse the placental barrier and access the fetus. An overview of the macaque maternal-fetal interface during the first trimester is presented in Fig. 6.

To our knowledge, this is the first study to document the outcome and histopathology of early pregnancy *L. monocytogenes* infection in a nonhuman primate model. From the present findings, we conclude that listeriosis in first trimester pregnancy leads to damage to decidual, placental, and fetal tissues followed by rapid fetal demise. In addition, the presence of bacteria within intervillous spaces (maternal circulation), villous capillaries, the umbilical cord, fetal liver, and fetal lungs (fetal circulation) is highly suggestive of a hematogenous route of fetal infection. Since it was not possible to determine the relative timing of decidual and placental infection in the present study, future studies tracing the spatiotemporal movement of *L. monocytogenes* through the nonhuman primate maternal-fetal interface will be useful in defining the pathogenesis of bacterial transmission to the fetus.

## MATERIALS AND METHODS

### Animals and breeding.

Adult female cynomolgus monkeys (*Macaca fascicularis*) were purchased from commercial vendors and maintained at the Wisconsin National Primate Research Center. All experimental procedures were performed in accordance with the NIH guidelines for care and use of laboratory animals and under the approval of the University of Wisconsin Graduate School Animal Care and Use Committee. Female monkeys were cohoused with compatible males and observed daily for menses and copulation. Pregnancy was detected by ultrasound examination of the uterus approximately 18 to 20 days following the predicted day of ovulation. The day of gestation when pregnancy was detected was estimated based on previous experience and published data describing cynomolgus macaque fetal size during gestation (31). Ultrasound examination of the uterus was done weekly or biweekly until the day of inoculation with *L. monocytogenes* and after inoculation on schedules described below.

### Inoculation with *L. monocytogenes*.

At varied days of gestation in the first trimester (days 36, 39, 40, and 46 [term is day 165]), monkeys were sedated, the uterus was examined by ultrasound to confirm a viable pregnancy, and approximately 10^7^ CFU log-phase cells of strain LM2203 (alternative designation, WS1) from a 2000 outbreak of listeriosis among pregnant women in Winston-Salem, NC (7), were administered in 10 ml of whipping cream via a soft intragastric feeding tube as previously described (32). Two monkeys were given a *Listeria*-free whipping cream inoculum in an identical manner at gestation days 38 and 41 and were included as uninfected controls for histological and physiological comparison. A portion of the inoculum was diluted, plated on blood agar, and incubated at 37°C to confirm precisely the CFU per milliliter of *L. monocytogenes* given to each animal.

### Fecal shedding.

Before and after inoculation, fecal samples were collected from cage pans for analysis of fecal shedding of *L. monocytogenes*. Schedules of sample collection are described in the legends to Fig. 1 and 2. Serial dilutions of fecal samples were plated in duplicate on modified Oxford agar plates (33), and the number of colonies was determined using ImageJ colony-counting software after 24 to 48 h of incubation at 37°C.

### Bacteremia monitoring.

Peripheral blood samples were collected periodically for aerobic and anaerobic culture to detect bacteremia as previously described (34) and processed on a BD Bactec 9050 blood culture system (BD Diagnostic Systems, Sparks, MD) in the Clinical Pathology Laboratory at the University of Wisconsin—Madison School of Veterinary Medicine. Aseptically inoculated pediatric Bactec Peds Plus/F blood culture bottles (BD Diagnostic Systems, Sparks, MD) were incubated until a positive signal was observed or for a maximum of 5 days. Bottles that were not positive at the end of 5 days were Gram stained and subcultured to verify the absence of bacterial growth.

### Surgery and tissue processing.

Ultrasound examination of the uterus was done 1 to 3 times per week after *L. monocytogenes* dosing to monitor fetal well-being and confirm fetal heartbeat and umbilical blood flow. When fetal demise was indicated by absence of heartbeat, fetal and maternal tissues were surgically collected at laparotomy. These were survival surgeries for the dams. For products of gestation, the entire conceptus (decidua, placenta, fetal membranes, umbilical cord, amniotic fluid, and fetus) was removed by uterotomy. Biopsy specimens of the placental bed (uterine placental attachment site containing deep decidua basalis, myometrium, and uterine serosa), maternal liver, spleen, and a mesenteric lymph node were collected aseptically. The fetus was dissected into 3- to 4-mm coronal segments, and alternating segments were fixed and embedded for histology (see below), or homogenized for bacteriological analysis on blood agar plates as previously described (35).

### Histology.

Dissected tissues were fixed in 2 to 4% paraformaldehyde for 24 to 72 h, rinsed in phosphate-buffered saline (PBS), and stored in 70% ethanol until processed and embedded in paraffin. Paraffin sections (5 μm) were stained with hematoxylin and eosin (H&E) and Gram stained using standard methods. Fetal trophoblasts in the decidua were visualized by chromogenic immunohistochemistry with anticytokeratin (CAM 5.2; 1 µg/ml [Becton, Dickinson]) using previously reported methods (36).

### Statistical analysis.

Analysis of the data for fecal and tissue CFU per gram was performed using GraphPad Prism version 5.0 (GraphPad Software, Inc., La Jolla, CA). For maternal vital signs and peripheral blood leukocyte data, means ± standard deviations (SD) were calculated for each group, and the statistical significance of the differences between pairs of groups was assessed, as well as the differences within individual preinfection versus postinfection measurements, using a mixed-effects logistic regression model with statistical consult at the UW Institute for Clinical and Translational Research.

10.1128/mBio.01938-16.5 TEXT S1  Supplemental materials and methods. Download TEXT S1, DOCX file, 0.01 MB. Copyright © 2017 Wolfe et al. 2017 Wolfe et al. This content is distributed under the terms of the Creative Commons Attribution 4.0 International license.
