# Supplementary material for: Acute Fetal Demise with First Trimester Maternal Infection Resulting from Listeria monocytogenes in a Nonhuman Primate Model
Source: mBio. 2017 Feb 21;8(1):e01938-16. doi: 10.1128/mBio.01938-16 (PMC5358912; doi:10.1128/mBio.01938-16)
Supplement: TABLE S1 [file mbo001173199st1.docx]

**Table S1. Maternal physiological responses to inoculation**

Reference intervals for adult female cynomolgus macaques:

Temperature: 95 - 100 ºF Systolic blood pressure: 84-130 mmHg

Mean arterial pressure: 81-113 mmHg Diastolic blood pressure: 53-80 mmHg

| **Subject & Lm**^a^ **dose** | **Gestation day** | **Post-Inoculation day** | **Weight (kg)** | **Rectal Temp ºF** | **Systolic Blood Pressure** | **Diastolic Blood Pressure** | **Mean Arterial Pressure** |
| --- | --- | --- | --- | --- | --- | --- | --- |
| **cy19** | 40 | 0 | 5.74 | 99.7 |  |  |  |
| 2.47 x 10^7^ | 48 | 8, sx^b^ |  | 102.1 |  |  |  |
|  | ----- | d5 post-sx |  | 96.5 |  |  |  |
|  | ----- | d12 post-sx |  | 98.7 |  |  |  |
| **cy21** | 36 | 0 | 3.35 | 100.1 | 131 | 77 | 96 |
| 1.23 x 10^7^ | 39 | 3 | 3.24 | 97.8 | 141 | 95 | 102 |
|  | 43 | 7 | 3.26 | 99.5 | 137 | 83 | 100 |
|  | 45 | 9 | 3.2 | 100.4 | 144 | 81 | 104 |
|  | 47 | 11 | 3.24 | 98.5 | 139 | 76 | 99 |
|  | 50 | 14, sx | 3.18 | 101.4 | 134 | 76 | 96 |
| **cy22** | 46 | 0 | 3.36 | 101.0 | 121 | 73 | 91 |
| 1.4 x 10^7^ | 50 | 4 | 3.25 | 99.6 | 110 | 58 | 77 |
|  | 53 | 7 | 3.26 | 101.0 | 120 | 63 | 83 |
|  | 56 | 10 | 3.29 | 99.7 | 126 | 71 | 83 |
|  | 60 | 14, sx | 3.32 | 101.1 | 132 | 71 | 92 |
| **cy25** | 39 | 0 | 3.32 | 100.5 | 130 | 73 | 93 |
| 0.9x10^7^ | 40 | 1 | 3.32 | 100.4 | 162 | 86 | 112 |
|  | 43 | 4 | 3.36 | 98.9 | 133 | 73 | 94 |
|  | 46 | 7 | 3.36 | 99.9 | 123 | 71 | 90 |
|  | 50 | 11, sx | 3.27 | 102.9 | 115 | 68 | 85 |
|  | ----- | d3 post-sx | 3.28 | 100.5 | 122 | 70 | 99 |
| **cy26**  No Lm | 38 | 0 (whipping cream) | 3.86 | 99.5 | 111 | 61 | 79 |
|  | 39 | 1 |  | 98.3 | 121 | 66 | 86 |
|  | 41 | 3 | 3.78 | 99.7 | 100 | 57 | 73 |
|  | 45 | 7 | 3.6 | 98.9 | 128 | 59 | 83 |
|  | 48 | 10 | 3.74 | 99.5 | 126 | 59 | 83 |
|  | 55 | 17, sx | 3.92 | 96.6 | 88 | 63 | 73 |
| **cy27**  No Lm | 41 | 0 (whipping cream) | 4.14 | 99.9 | 118 | 61 | 81 |
|  | 42 | 1 |  | 99 | 115 | 59 | 79 |
|  | 44 | 3 | 4.06 | 99.5 | 133 | 37 | 70 |
|  | 48 | 7 | 4.01 | 99 | 107 | 58 | 76 |
|  | 51 | 10, sx | 4.12 | 98.1 | 92 | 38 | 57 |

^a^*Listeria monocytogenes* ^b^surgical collection of conceptus and maternal biopsies.

A blank cell indicates no data was available.
