# Supplementary figures and images for: Acute Fetal Demise with First Trimester Maternal Infection Resulting from Listeria monocytogenes in a Nonhuman Primate Model
Source: mBio. 2017 Feb 21;8(1):e01938-16. doi: 10.1128/mBio.01938-16 (PMC5358912; doi:10.1128/mBio.01938-16)

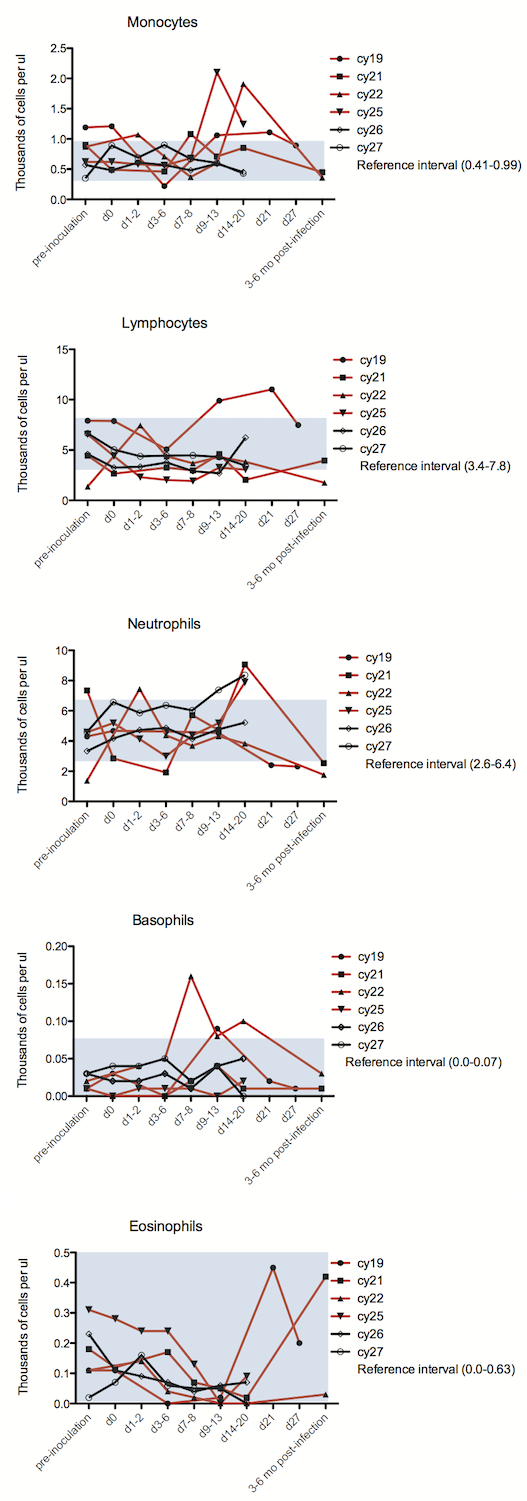

Supplement: FIG S1 [file mbo001173199sf1.tif]

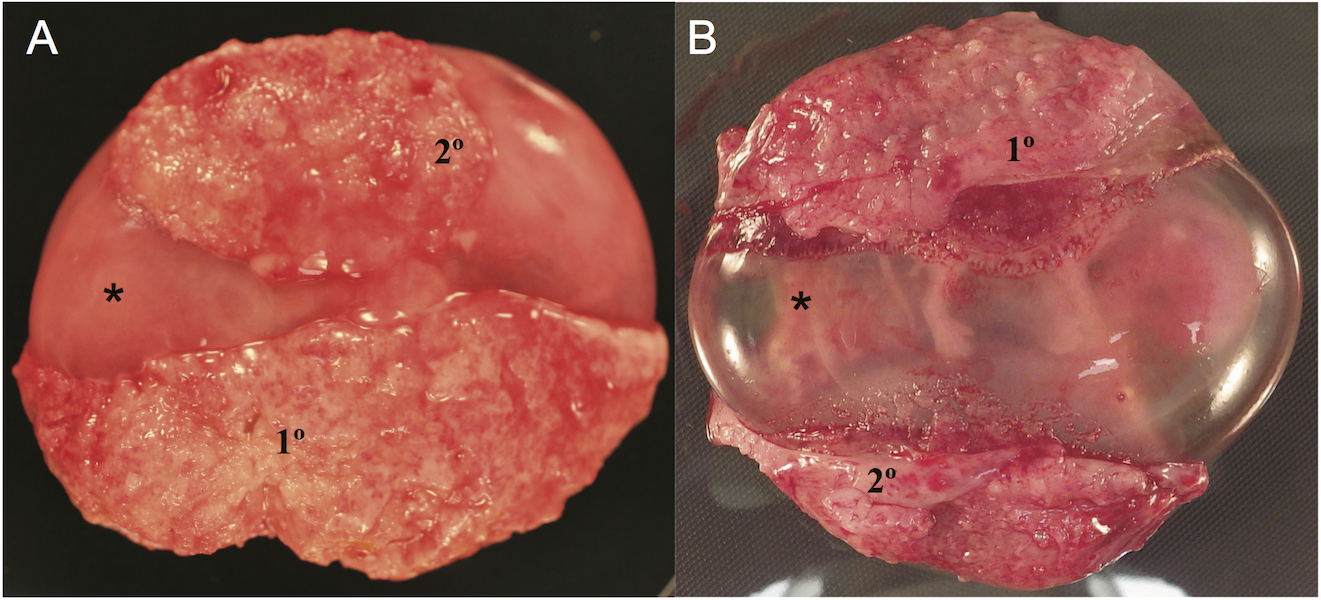

Supplement: FIG S2 [file mbo001173199sf2.tif]

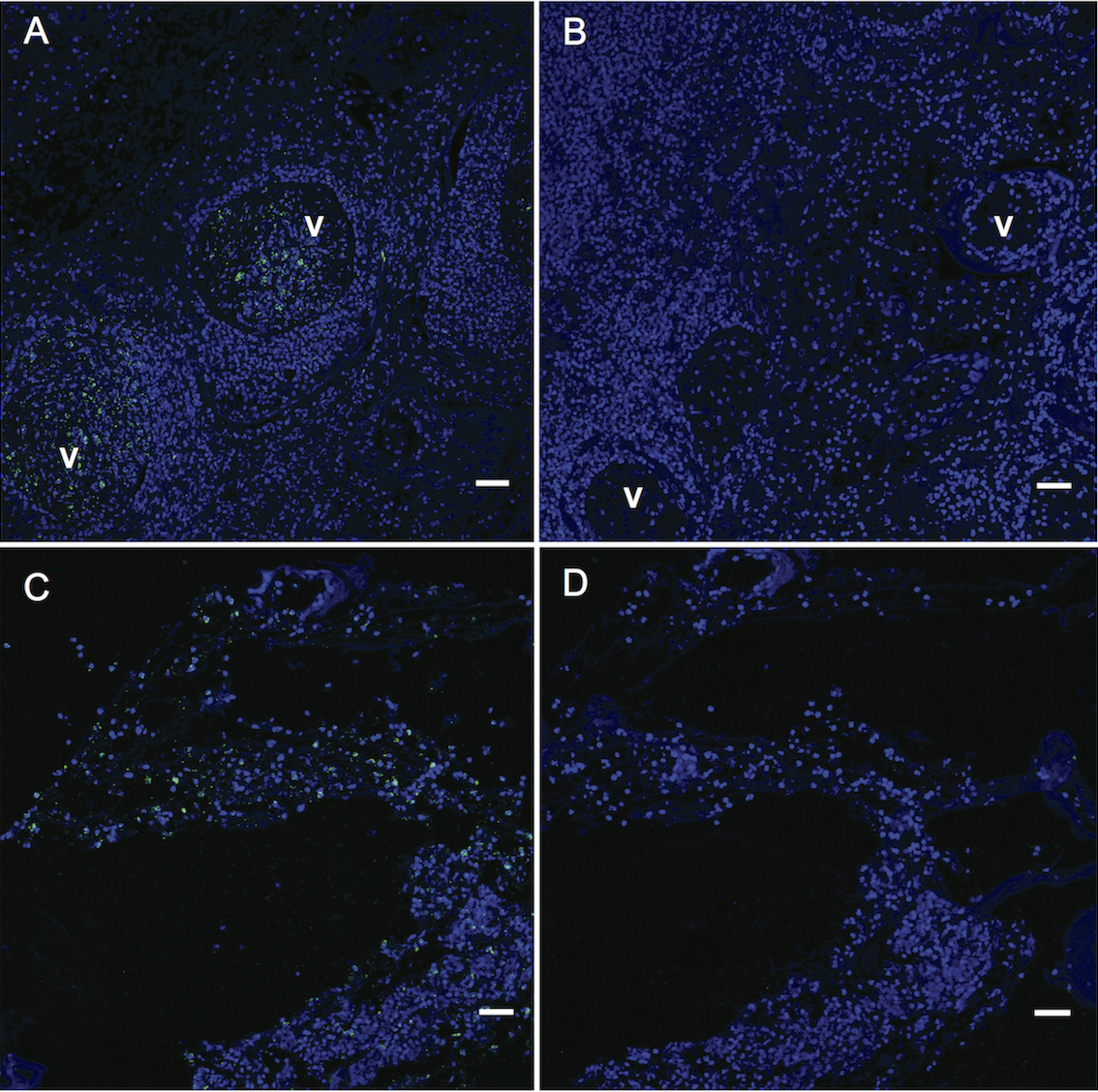

Supplement: FIG S3 [file mbo001173199sf3.tif]
