# Supplementary material for: Acute Fetal Demise with First Trimester Maternal Infection Resulting from Listeria monocytogenes in a Nonhuman Primate Model
Source: mBio. 2017 Feb 21;8(1):e01938-16. doi: 10.1128/mBio.01938-16 (PMC5358912; doi:10.1128/mBio.01938-16)
Supplement: TEXT S1 [file mbo001173199s1.docx]

**SUPPLEMENTAL MATERIALS & METHODS**

The presence of *L. monocytogenes* in various tissues was confirmed by fluorescent immunohistochemistry with Listeria O antiserum (Difco Laboratories’ rabbit polyclonal antibody against the somatic O antigen of Listeria serogroup 4, 1:500). Briefly, deparaffinized tissue sections were boiled in 10mM sodium citrate buffer at pH 6.0 for epitope retrieval, incubated with blocking buffer (PBS, 1% BSA, 5% donkey serum) for 30 minutes, incubated overnight at 4°C with the primary antibody, and incubated for 1 hour with an Alexa Fluor 488-conjugated secondary antibody (anti-rabbit igG donkey, 1:500). Nuclei were stained with DAPI, slides were cover-slipped using Vectashield mounting medium, and examined on a Nikon A1 confocal microscope.
